# Bridging health and global climate policy: Opportunities for policymakers and health systems

**DOI:** 10.1016/j.joclim.2025.100607

**Published:** 2025-11-14

**Authors:** Pranay Narang, Arianne Teherani

**Affiliations:** a University of California, Berkeley - University of California, San Francisco Joint Medical Program, California, United States of America; b University of California, School of Medicine, San Francisco, California, United States of America; c University of California, Center for Climate, Health and Equity, California, United States of America

**Keywords:** Nationally determined contributions, Decarbonization strategies for health systems, Recommendations for the healthcare sector, Recommendations for policymakers

## Abstract

Health is increasingly recognized as a priority within global climate policy yet remains underrepresented in instruments such as Nationally Determined Contributions (NDCs). The 2025 NDC Revision Cycle, ahead of COP30, presents a critical opportunity to operationalize existing health-related recommendations by integrating measurable, health-focused commitments into NDCs. This article proposes a set of actionable commitments aligned with broader priorities to mobilize health-specific climate finance, phase out fossil fuels, integrate climate-health indicators, advance health-related mitigation and adaptation strategies, and synchronize with national health strategies, which are suitable for adoption in the 2025 NDC revisions and future policy processes.

In parallel, we outline how the healthcare sector can accelerate uptake of these health-focused commitments and advance national mitigation and adaptation goals by: (1) strengthening climate-health data infrastructure, (2) establishing multi-level platforms that connect health stakeholders with policymakers, and (3) implementing decarbonization strategies.

By embedding health-focused commitments into NDCs and positioning healthcare systems as data providers and decarbonization leaders, these measures can elevate the health sector’s influence on climate policy, enhance real-time awareness of health risks, and mobilize climate financing. We situate these recommendations within the live policy window of the 2025 NDC revision cycle, the 2028 Global Stocktake, and persistent financing gaps to foster more informed, coordinated engagement by the health sector in shaping global climate commitments.

## Highlights

• NDCs must integrate measurable health commitments to accelerate policy progress.
• Health systems must report climate-health data to influence climate policy.
• Health systems must drive decarbonization to advance mitigation and adaptation goals.

## 1 Introduction

2023 marked a decisive shift in global climate policy, when health was formally recognized as an essential consideration in future climate negotiations, strategies, and policies – a focus reaffirmed at Conference of the Parties (COP)29, where world leaders recognized the climate crisis as not only an environmental emergency but also as an urgent public health imperative [1]. Yet progress on COP29’s core priority – negotiating the total annual financing that wealthier nations will collectively mobilize to support vulnerable countries in mitigation, adaptation, and loss and damage – fell short [2,3]. Countries agreed to increase the current $100 billion target to $300 billion [4], a step forward but still a fraction of the estimated $1 trillion per year in external finance needed by 2030 and the broader $1.3 trillion annual goal by 2035 [5,6]. This shortfall undermines the ability of low-and-middle-income-countries (LMICs) to scale emissions reductions and strengthen adaptation efforts.

Now, in 2025, countries are revising their Nationally Determined Contributions (NDCs) – self-defined pledges outlining emission reduction and adaptation strategies – ahead of COP30. Instituted under the Paris Agreement, NDCs are key policy instruments shaping global climate ambition, financing, and domestic implementation [7,8]. Sectors such as energy, agriculture, and land use have long recognized NDCs as powerful levers to secure political commitments, mobilize financing, and shape national policy agendas by proactively embedding their priorities into NDCs [9].

Despite the escalating toll of heat-related illness, vector-borne diseases, and disrupted health services, health remains underrepresented in most NDCs. One study found that only 60 countries (32 %) mention health in their NDCs, and just 40 include detailed health adaptation plans as of 2021 [8]. As of February 2024, only 47 % of 58 updated NDCs refer to health, suggesting that engagement may be further declining [[8], [10], [11], [12]].

The 2025 NDC revisions present a critical opportunity to embed health meaningfully into global climate commitments. Doing so offers several benefits, including addressing societal health needs, raising awareness of climate-health risks, highlighting the human costs of inaction, accelerating investment in health system resilience, and aligning national adaptation efforts with public health needs [13]. Integrating health into NDCs and future policy frameworks will help ensure governments are held accountable to the real-time consequences of delayed climate action.

Furthermore, revised NDCs will increasingly shape the regulatory, financial, and infrastructural landscape in which health systems operate. It is therefore imperative that healthcare stakeholders understand how their daily work intersects with broader climate policy and are prepared to respond, adapt, and lead accordingly.

To these ends, this article aims to:

(1). Provide actionable guidance for policymakers to integrate measurable, health-focused commitments into the 2025 NDC revision cycle and future global climate policy processes.
(2). Equip the healthcare sector with strategies to respond to the evolving policy landscape and support the adoption of these health-focused commitments.

## 2 Bridging global climate policy and health systems

### 2.1 What we wish to see from policymakers – integrating health into global climate policy

The revised 2025 NDCs will directly influence higher-level policy processes, including the 2028 Global Stocktake (GST), a key accountability mechanism under the Paris Agreement where representatives from 198 countries “take stock” of their collective progress and recalibrate commitments. The first GST, conducted at COP28, concluded that current commitments fall short of the Paris Agreement’s goals. The upcoming GST will determine long-term priorities, policies, and strategies, with the 2025 NDC cycle informing that trajectory.

Recognizing the persistent underrepresentation of health in NDCs, the World Health Organization (WHO), Global Climate and Health Alliance (GCHA), the Lancet Countdown, and leading civil society coalitions have issued frameworks addressing this gap [10,12,14]. Across these frameworks, there is strong alignment on several recommendations, including (1) explicitly embedding health in both mitigation and adaptation components of NDCs; (2) recognizing and quantifying the health co-benefits of mitigation strategies (e.g. air quality, sustainable diets, active transport); (3) prioritizing protection for vulnerable populations; (4) strengthening health system adaptation through resilient infrastructure, service continuity, emergency preparedness, and workforce training; (5) incorporating climate-health indicators for monitoring and early warning; (6) establishing health-specific climate finance mechanisms which are dedicated funding streams sourced from public, private, or multilateral channels earmarked for mitigation and adaptation activities within the health sectors; and (7) creating inclusive, cross-sectoral governance that integrates health ministries into climate decision-making.

These frameworks offer a clear roadmap, yet analyses illustrate that few countries have operationalized these recommendations into tangible, time-bound commitments [[8], [10], [11], [12]]. To address this gap, and in preparation for COP30 and the 2028 GST, below we propose measurable, health-focused commitments aligned with these recommendations and suitable for modification and integration into the 2025 NDC revision cycle and future global climate policy (Table 1) [10]. In parallel, we outline how the health sector can support and accelerate adoption of these commitments (Section 2.2).

**Table 1:** Proposed commitments for inclusion in NDCs.

Proposed Commitments for Inclusion in NDCs
**Mobilize Health-Specific Climate Financing** Establish dedicated public and private funding streams for health-focused mitigation and adaptation efforts, including strengthening health system resilience, workforce capacity, disease surveillance, and protecting vulnerable populations, with clear mechanisms for allocation tracking and an explicit annual target for health-focused financing. Require annual public reporting of allocations and disbursements, and the inclusion of these funds as a distinct, measured category in national climate finance tracking systems [12,14].
**Fossil Fuel Phase Out as a Health Imperative** Commit to phasing out fossil fuel exploration, extraction, and infrastructure expansion and transitioning to renewable energy by setting a national phase-out schedule, milestones, and an annual target of deploying at least 1.5 Terawatts (TW) of renewable energy capacity additions (wind, solar, and geothermal). Explicitly exclude gas as a transition fuel and reliance on biomass/ammonia burning, carbon capture and storage, and other measures that extend fossil fuel dependency and drive environmental degradation [15,16].
**Advance Health-Focused Mitigation & Adaptation Strategies** Require all major public and private healthcare organizations to measure and report full-scope (Scopes 1–3) greenhouse gas (GHG) emissions. At present, no universally accepted standards exist for measuring emissions, but such data reporting can guide national policy and incentivize further reductions. Identify a national target year for net-zero health sector emissions with interim milestones. Require health impact assessments for all major climate policies and infrastructure projects to identify risks such as mortality, morbidity, and health system disruptions; and develop a national climate-health adaptation plan informed by local vulnerability mapping and mental health risks assessment [17].
**Embed Climate & Health Indicators & Resilience Targets** Integrate a set of core climate change and health indicators (CCHIs) into NDC monitoring systems. These indicators should quantify multi-directional links between climate events and human health, covering mortality, morbidity, and service disruption from direct and indirect climate impacts (e.g. heatwaves, floods, wildfires, droughts, ecosystem alterations, crop failures, reduced marine food capture, zoonotic spillovers) [18]. Use CCHIs to support early warning systems, identify at-risk populations, track progress, and foster consistent health-related communication between policymakers and health stakeholders. In parallel, establish resilience benchmakers for all hospitals and primary health centers in risk-prone regions to ensure resilient supply chains and equitable access to services during and following disasters [[19], [20], [21], [22], [23], [24]].
**Synchronize NDCs with National Health Policies** Align NDC health commitments, targets, timelines, and associated economic considerations with national health strategies. Establish multisectoral climate-health coordination mechanisms, such as national platforms with health sector representation, to design and implement climate-health strategies. Create cross-ministerial working groups involving health, environment, and climate change departments to conduct joint assessments of climate impacts on health, align timelines and review cycles for NDCs and national health plans, foster joint implementation, and coordinate resilience planning.

These commitments should be accompanied with implementation schedules adapted to each country’s context, capacity, and policy environment.

### 2.2 What we wish to see from the health sector

While policy reform is essential, the success of integrating health into NDCs and related processes ultimately depends on health systems’ ability to convey the immediate health impacts of climate change while advancing national mitigation and adaptation goals. This requires establishing climate-health data infrastructure, building platforms to bridge frontline perspectives and climate-health evidence with policymakers, and transitioning health systems toward low-carbon operations.

#### 2.2.1 Strengthening and leveraging climate-health data infrastructure to inform policy

First, health systems should establish mechanisms for collecting, analyzing, and reporting climate-related health outcome data, including tracking the prevalence of climate-sensitive diseases (e.g. asthma, heatstroke, vector-borne diseases, etc.), resource utilization during disasters (e.g. hospital admissions, emergency services demand), and the effectiveness of public health interventions (e.g. heatwave response plans, vaccination campaigns for vector-borne diseases, etc.). Systematically gathered, such data can feed into and inform future NDC development, national adaptation planning, and policy processes, ensuring health considerations are grounded in timely evidence.

To accomplish this, health systems must expand climate health surveillance capacity, modeled after existing systems for infectious disease monitoring. These platforms should integrate climate-health indicators with environmental and public health data. Examples include heat-related illnesses, healthcare delivery disruptions, and disease trends. Regularly updated and shared with local, state, and national authorities, these data would inform real-time decisions, resource allocation, and emergency response [25]. In doing so, surveillance systems enable policies that are not only reactive to crises but also proactive in addressing long-term climate and health risks.

Furthermore, to proactively amplify the health sector’s influence on climate policy, health systems must participate in multilevel, collaborative platforms that convene healthcare providers, data scientists, community leaders, and policymakers. For instance, Australia’s National Health and Climate strategy established governance structures linking federal, state, academic, and community stakeholders, while India’s Public Health Foundation of India, under the Ministry of Health, coordinates efforts to integrate climate resilience across state health departments, health facilities, and ministry-level policy [26,27]. Such platforms elevate health data from clinical and community settings to policymakers, ensuring on-the-ground perspectives and health evidence informs future NDC revisions, adaptation priorities, and financing decisions.

#### 2.2.2 Driving health system decarbonization to advance national climate commitments

Health systems leaders can advance decarbonization by aggregating and analyzing emissions data annually, setting emissions targets, reducing energy use and waste, and publicly reporting on progress for their own environmental footprint [28]. Institutional mechanisms should include sustainability leadership, incentive structures tied to sustainability progress [29], and alignment with national NDC targets to ensure transparency and enable sectoral progress tracking. Since supply chain activities account for over 80 % of total emissions, transitioning to sustainable procurement and operations is essential [30,29]. By integrating sustainability criteria into purchasing decisions, the healthcare sector can leverage their significant market power to influence suppliers to adopt lower-emission practices. In doing so, the healthcare sector can set a benchmark for adjacent industries like manufacturing, transportation, and energy to follow [31]. Insurance can further accelerate this shift by incentivizing low-emission practices and climate-conscious programs, as Medicare and Medicaid have already begun piloting in the US [32].

Several countries offer lessons for decarbonization strategies. In the United States (U.S.), more than 1200 hospitals – representing over 19 % of U.S. hospitals – have signed the *Health Sector Climate Pledge*, committing to reducing emissions 50 % by 2030 and achieving net zero emissions by 2050 [33]. These institutions pledge to publish annual progress, conduct supply chain emissions inventories, and develop resilience plans for vulnerable communities. In the United Kingdom (U.K.), the National Health Service (NHS) has committed to becoming the world’s first net-zero health system, supported by emissions tracking, green procurement policies, and carbon literacy training for clinical staff.

Accreditation bodies can play a critical role in scaling health sector decarbonization. In the U.S., the Joint Commission has launched a sustainability certification program that provides resources to support hospitals in transitioning to more sustainable operations. To ensure lasting impact, decarbonization progress should be incorporated into the Joint Commission’s reaccreditation process. In the U.K., the NHS has tied carbon performance to financial and operational incentives. Embedding sustainability metrics into accreditation frameworks can institutionalize long-term accountability and align healthcare operations with national climate commitments and NDC goals.

Most LMICs face cost barriers that limit their ability to implement such strategies, renewable energy transitions, and sustainable procurement. Equitable financing mechanisms, negotiated at future COP summits, will be critical to strengthen infrastructure, build capacity, and ensure sustainable healthcare delivery globally.

## 3 Conclusion

Without urgent, coordinated action between policymakers and the health sector, the climate crisis threatens to undo the last 50 years of progress in global health, development, and poverty reduction. Health shocks and stresses already push around 100 million people into poverty each year, and climate change will sharply accentuate this trend [34]. The health sector has lagged behind others in shaping climate policy. With NDC revisions underway ahead of COP30, this article proposes key health-focused commitments for integration into NDCs and highlights how health systems can support their adoption while simultaneously advancing mitigation and adaptation goals.

## CRediT authorship contribution statement

**Pranay Narang:** Writing – review & editing, Writing – original draft, Investigation, Formal analysis, Conceptualization. **Arianne Teherani:** Methodology, Investigation, Conceptualization, Supervision.

## Declaration of competing interest

The authors declare that they have no known competing financial interests or personal relationships that could have appeared to influence the work reported in this paper.
